# Minimally Invasive Excision of Subchondral Osteoid Osteoma Using O-Arm Navigation and Arthroscopic Visualization

**DOI:** 10.7759/cureus.96938

**Published:** 2025-11-16

**Authors:** Mohamed A Alawadhi, Abdulla Aljowder, Abdulrahman D Mohroofi, Mohammed Al Tamimi, Osama A Zeidan

**Affiliations:** 1 Pediatrics, Royal Medical Services, Manama, BHR; 2 Orthopedic Surgery and Sports Medicine and Arthroplasty, Royal Medical Services, Muharraq, BHR; 3 Pediatrics, King Hamad University Hospital, Manama, BHR; 4 Radiology, Royal Medical Services, Muharraq, BHR; 5 Orthopedics, Royal College of Surgeons in Ireland - Bahrain, Busaiteen, BHR

**Keywords:** arthroscopic visualization, dual-modality approach, novel, o-arm navigation, osteoid osteoma

## Abstract

Osteoid osteoma is the third most common benign bone tumor, composed of osteoid and woven bone that rarely exceeds 1.5 cm in dimension. The typical presentation is intense pain that mostly worsens at night and is relieved with non-steroidal anti-inflammatory drugs. X-ray is the initial imaging modality; however, CT scan is the modality of choice as it can identify almost all cases of osteoid osteoma. Our case demonstrates that the integration of O-arm navigation with arthroscopic visualization offers a novel and effective approach for managing subchondral osteoid osteomas. Although traditional surgical excision and CT-guided radiofrequency ablation have shown long-term high success rates, they are associated with more morbidity and cosmetically undesirable scarring. Our technique combines the benefits of both approaches while minimizing their respective limitations. This dual-modality approach proved particularly valuable in our young, active patient, where preservation of joint integrity and rapid return to function were paramount. The successful outcome suggests that this technique may represent a significant advancement in the surgical management of subchondral osteoid osteomas.

## Introduction

Osteoid osteoma is a benign skeletal neoplasm composed of osteoid and woven bone that rarely exceeds 1.5 cm in dimension [1]. However, its pathogenesis remains debated, with some investigators considering it a benign neoplasm of osteoblast origin (given its histologic resemblance to osteoblastoma) and others favoring an inflammatory etiology due to the relatively small size, self-limited nature, and occasional presence of intracellular viral particles [2,3].

Pathophysiologically, prostaglandins increase the vessels’ diameter and permeability, raising both the vessels’ size and the flow, leading to higher pressure and pain. Prostaglandins also amplify the pain by affecting the bradykinin system [4]. Furthermore, the response to non-steroidal anti-inflammatory drugs (NSAIDs), which affect prostaglandin synthesis, supports the fundamental role of prostaglandins in pain generation [2,5]. The diagnosis is usually established based on its characteristic clinical and imaging features.

Accounting for 10-14% of all benign bone tumors and 2-3% of all primary bone tumors, osteoid osteoma is the third most common benign tumor of bone [3]. Approximately 70% of cases are diagnosed during the second decade of life, with a male-to-female ratio ranging between 2:1 and 3:1 [2,6]. Classification depends on the lesion’s anatomical location, with intracortical lesions representing about 75% of cases, frequently involving the diaphysis (approximately 50%), followed by the metaphysis (around 40%) [6]; cancellous osteoid osteomas accounting for nearly 20%. Subperiosteal lesions are the least common (around 5%), with subchondral lesions being particularly rare.

Clinically, the typical presentation of extra-articular osteoid osteoma includes severe pain often worsening at night in up to 80% of cases, which is relieved by aspirin or NSAIDs [1,6]. In contrast, intra-articular lesions, though less common (with fewer than 15% occurring intra-articularly and only 1.2% to 2.7% affecting the pelvis, primarily in patients younger than 30 years) [7,8], may present with joint effusion, synovitis, restricted range of motion, and other signs related to local inflammation. Spinal lesions may lead to painful scoliosis or, when localized in the cervical region, cause torticollis with reduced neck mobility [3,9]. Brodie abscess, chondroblastoma, osteoblastoma, and osteosarcoma can mimic osteoid osteoma and are considered differential diagnoses to be distinguished by radiological and histological findings [10].

Given the challenging localization and the risk of damaging the chondral surface (especially for subchondral lesions of the femoral head), recent surgical innovations have been directed toward minimally invasive techniques. This report aims to describe a novel and minimally invasive surgical approach using the O-arm navigation in conjunction with arthroscopic visualization.

## Case presentation

An 18-year-old male presented to the orthopedic clinic with a four-month history of lower back and pelvic region pain. The pain was described as stabbing in nature and radiated to the superior border of the right patella. The pain was aggravated by jogging activities and at night. The patient used NSAIDs that mildly relieved the pain. He denied a history of trauma or falls.

On examination, tenderness was noted over the right posterior superior iliac spine. The right hip’s range of motion was restricted to 30-degree flexion due to pain. All other tests, including the neurovascular and FABER tests, were unremarkable. The physical examination and patient history were consistent with osteoid osteoma. A pelvic MRI was performed to rule out serious pathology, which revealed a well-defined subchondral lesion in the right femoral head with low T1 signal, high T2 signal with dark sclerotic rim, and internal tiny foci suggesting a nidus. Surrounded by bone marrow edema reaching to the intertrochanteric region. Findings supported the diagnosis of osteoid osteoma. Physiotherapy and NSAIDS (Celebrex) were recommended for pain management.

**Figure 1:**
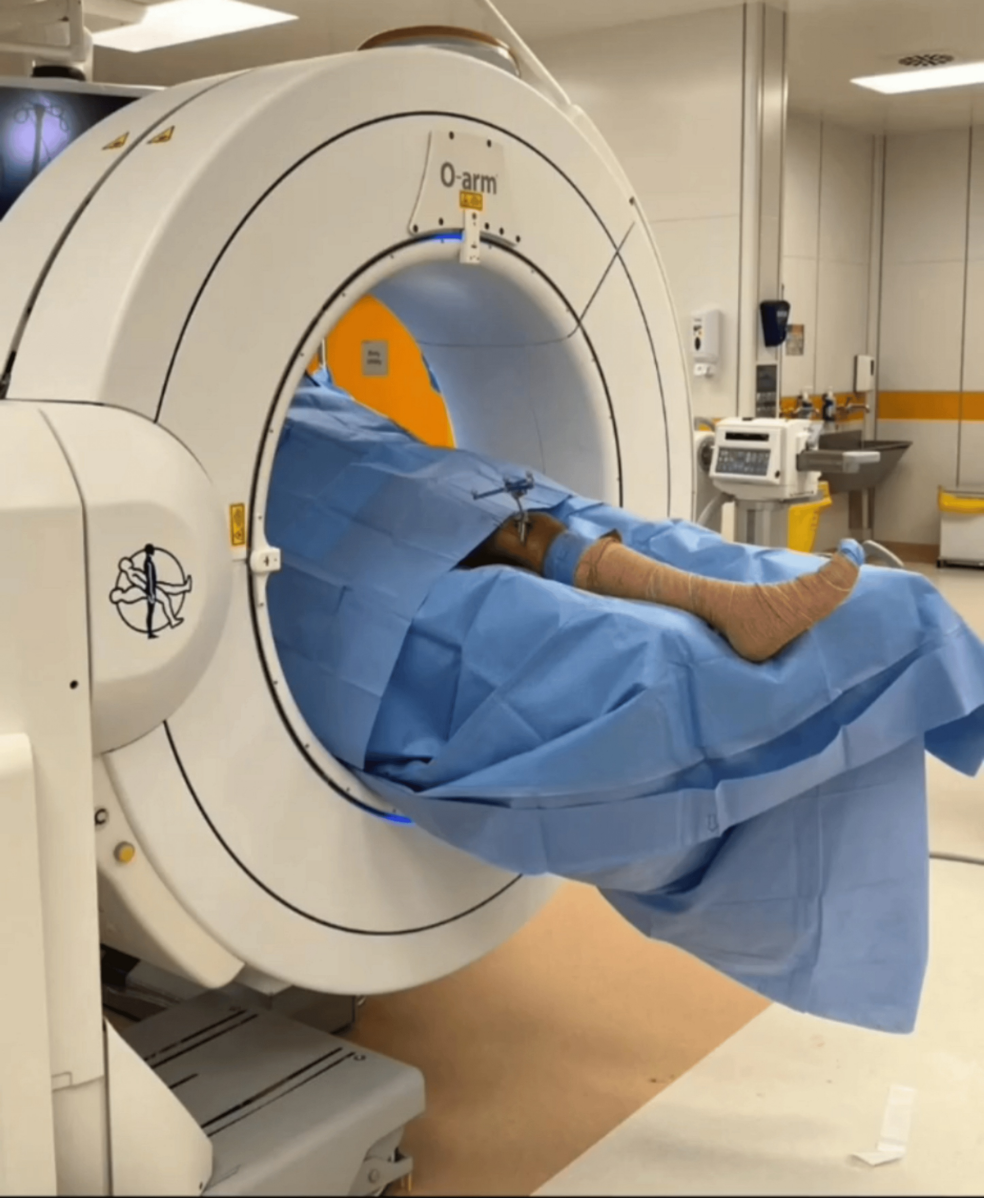
Intraoperative image of the patient’s position with O-arm imaging.

Our case utilized a novel surgical approach that integrates O-arm navigation with arthroscopic visualization to enhance precision and minimize collateral damage. The patient was positioned on the operating table with the O-arm correctly aligned (Figure 1).

**Figure 2:**
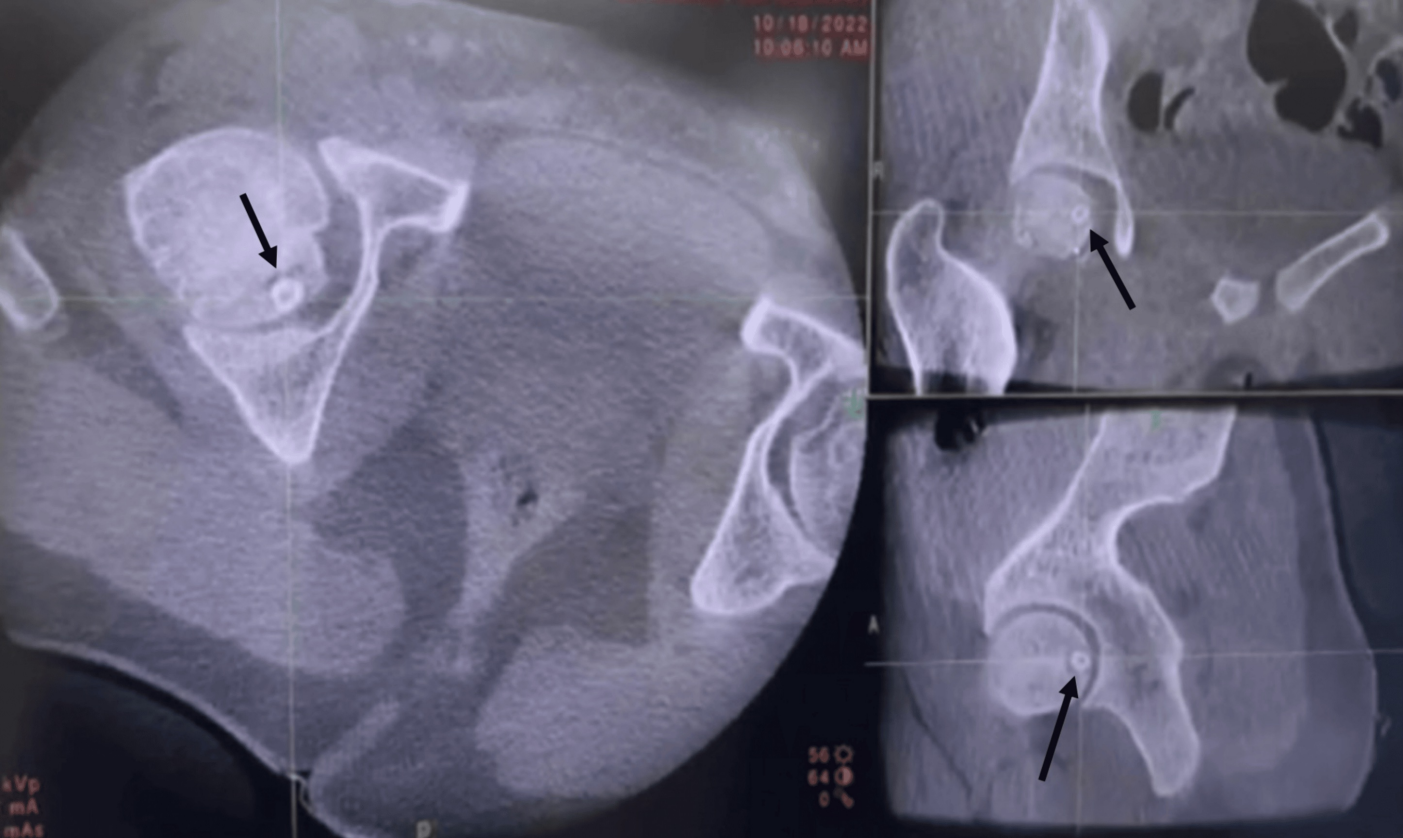
Three-dimensional O-arm imaging, with black arrows pointing toward the lesion.

The O-arm system provided real-time, three-dimensional imaging, which was displayed on the monitor (Figure 2), enabling an accurate localization of the subchondral osteoid osteoma.

This advanced imaging modality significantly improves the surgeon’s ability to determine the optimal drilling trajectory, thereby reducing the risk of inadvertent injury to critical structures such as the chondral surface.

**Figure 3:**
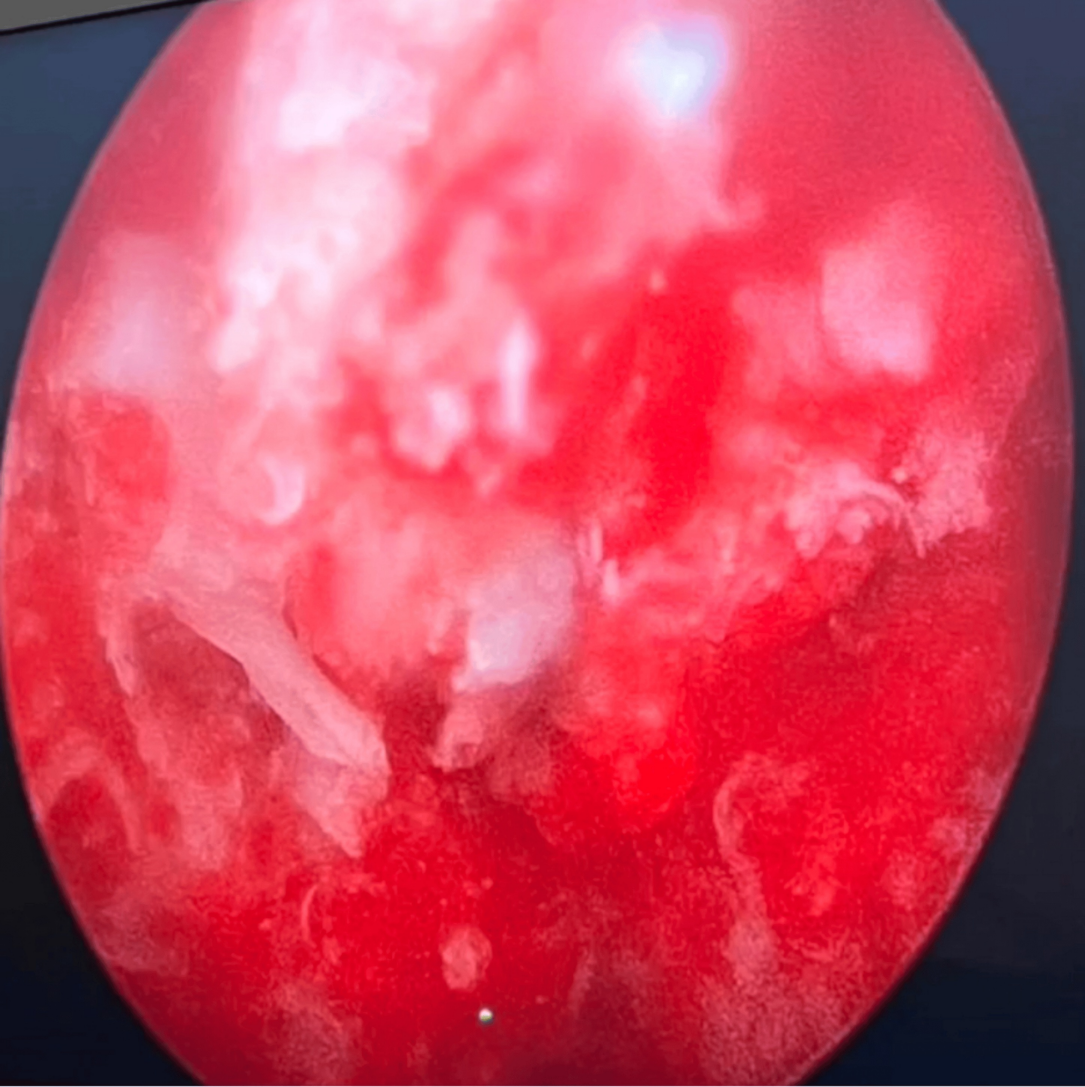
Arthroscopic image of the subchondral hypervascular nidus.

After establishing the correct trajectory using the O-arm navigation system, the patient, positioned supine on a radiolucent table with the limb in neutral rotation, underwent creation of an anterolateral arthroscopic portal approximately 2 cm anterior and 1 cm distal to the tip of the greater trochanter. Through this portal, a 30-degree 4.0-mm arthroscope was introduced via the bone tunnel under navigational guidance, allowing direct visualization of the lesion. Arthroscopic assessment confirmed the presence of the nidus, which exhibited the characteristic hypervascular and reddish appearance (Figure 3). The nidus was meticulously curetted under direct visualization while preserving the surrounding subchondral bone. Following excision, 10 mL of 6% hydrogen peroxide was instilled for approximately 30-45 seconds for effervescent cleansing and hemostasis, followed by thorough flushing with normal saline to prevent chemical irritation. Arthroscopic reassessment confirmed complete removal of the nidus and integrity of the subchondral plate.

**Figure 4:**
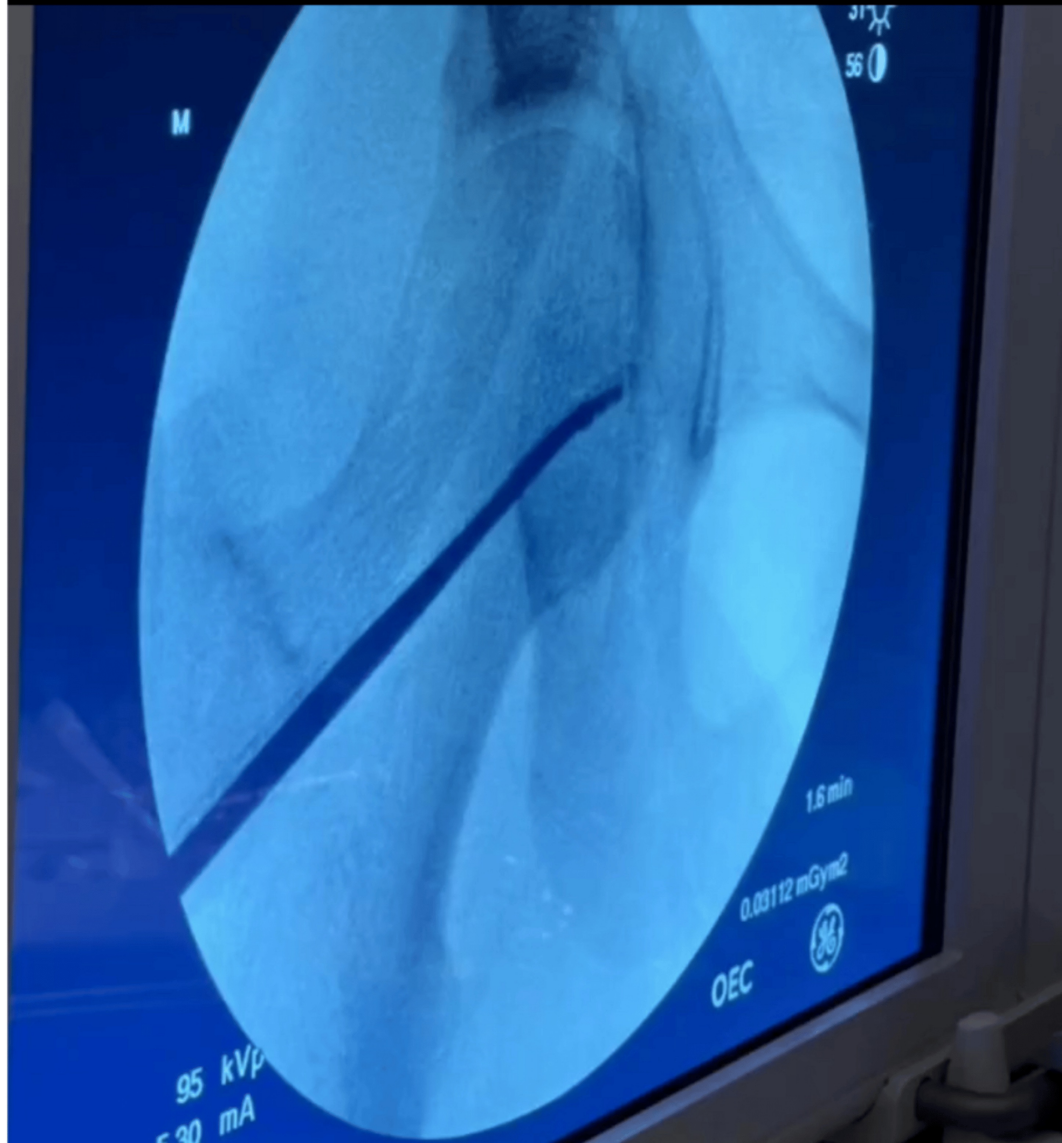
X-ray of the right femoral head while using the curettage instrument on the subchondral area.

During the procedure, fluoroscopic guidance was employed to direct the curettage process (Figure 4), ensuring that the lesion was carefully excised while preserving the integrity of the adjacent subchondral bone.

**Figure 5:**
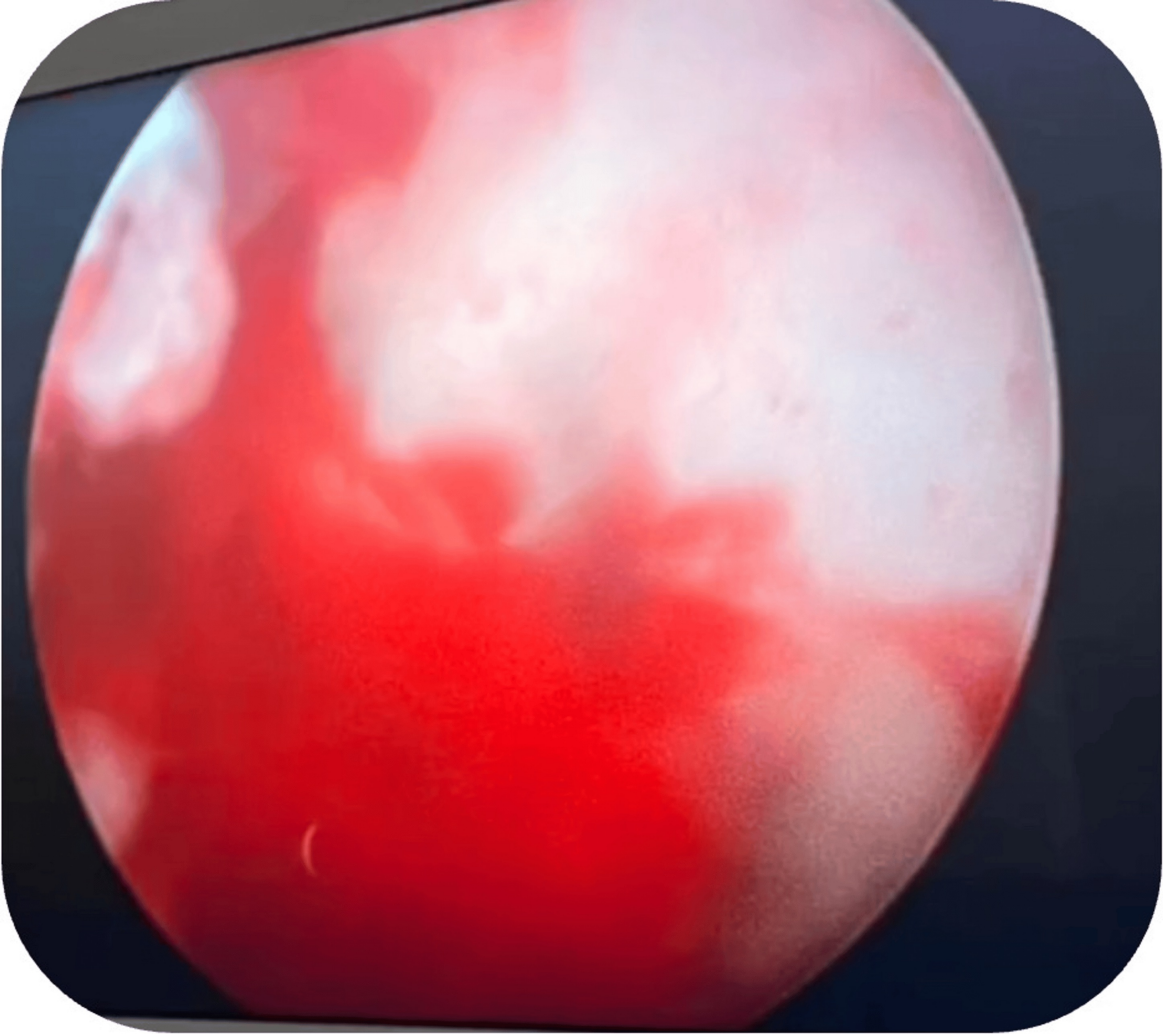
Subchondral bone visualization via the arthroscope after excision of the lesion.

Once the nidus was completely removed, a final arthroscopic inspection (Figure 5) confirmed adequate debridement and the preservation of the subchondral plate.

Post-excision, O-arm showed no remnant of the nidus. Histopathology section revealed bony tissue with a circumscribed nidus of anastomosing trabeculae of woven bone rimmed by plump osteoblasts in a highly vascular background. The characteristics were consistent with a clinical diagnosis of an osteoid osteoma. Preoperatively, the patient’s Visual Analog Scale score improved to 1/10 at six weeks. Functionally, the Harris Hip Score improved from 62 to 95, with the patient regaining a full range of motion and returning to unrestricted physical activity.

The postoperative CT scan showed a healed cortical tunnel from the curettage tract, which radiologically resembled a post-ablation track; however, no radiofrequency ablation was performed in this case. Postoperatively, partial weight-bearing (approximately 50%) was permitted for two weeks, progressing to full weight-bearing by week six. Gentle range of motion exercises were initiated on postoperative day one, allowing early recovery and return to function.

## Discussion

The management of osteoid osteoma, particularly in subchondral regions, presents distinct diagnostic and therapeutic challenges that require a multimodal approach. Initial imaging with X-ray is commonly utilized in evaluating bone pain and, when combined with clinical history, demonstrates an accuracy of approximately 75% [11,12]. On radiographs, the characteristic appearance of a radiodense central nidus surrounded by a halo of reactive sclerosis is seen in most cases [1]. However, the intensity of the sclerotic reaction is highly dependent on the lesion’s anatomical location. While diaphyseal lesions show prominent sclerosis, subchondral and subperiosteal osteoid osteomas may exhibit little to no reactive sclerosis [9].

Advanced imaging plays a critical role in preoperative planning. Although MRI is indispensable for evaluating soft tissue changes, its sensitivity is compromised by the possibility of missing the nidus in up to 35% of cases [12,13]. In contrast, CT remains the imaging modality of choice due to its near-perfect sensitivity in identifying the nidus and its surrounding bony architecture [14]. These imaging characteristics are pivotal in accurately localizing the lesion and planning a safe surgical corridor.

Historically, osteoid osteoma is known as a self-limiting condition with reports of spontaneous regression within 6-15 years with the use of NSAIDs [15,16]. Nonetheless, such a conservative approach is less desirable in young, active patients who demand a rapid restoration of function. Traditional open surgical resection, while achieving symptomatic relief with an 88-97% long-term success rate, is associated with higher morbidity, prolonged hospital stays, and cosmetically undesirable scarring [10]. Percutaneous image-guided techniques, such as radiofrequency ablation, have emerged as less invasive alternatives, with a clinical success rate exceeding 90% [17,18]. However, in subchondral lesions, radiofrequency ablation risks causing thermal injury to the articular cartilage. The differential diagnosis for this presentation included osteoblastoma and Brodie’s abscess. Osteoblastoma was excluded based on lesion size (<1.5 cm) and characteristic nocturnal pain responsive to NSAIDs. Brodie’s abscess was ruled out due to the absence of systemic infection signs and the presence of a well-defined radiolucent nidus with surrounding sclerosis, typical of osteoid osteoma.

Our combined approach of O-arm navigation and arthroscopic visualization offers distinct advantages. It allows for accurate intraoperative localization, minimizes the risk of damaging the chondral surface, and ensures complete lesion removal while preserving the subchondral bone. Furthermore, the integration of hydrogen peroxide 6% irrigation post-curettage provides an additional measure of cleaning and reduces residual cellular material, thereby mitigating recurrence risks. The postoperative outcome was excellent, with the patient reporting complete pain resolution and early return to physical activities.

Histopathological examination revealed bony tissue with a circumscribed nidus composed of anastomosing trabeculae of woven bone, rimmed by plump osteoblasts in a highly vascular background, confirming the diagnosis of osteoid osteoma.

In summary, the present case reinforces that the integration of advanced imaging modalities, specifically O-arm navigation, with arthroscopic techniques can overcome the limitations of traditional surgery and percutaneous ablation when managing subchondral osteoid osteoma. This novel minimally invasive strategy optimizes the balance between effective lesion excision and the preservation of joint integrity, ultimately leading to improved clinical outcomes in demanding patient populations.

## Conclusions

Our case demonstrates that the integration of O-arm navigation with arthroscopic visualization offers a novel and effective approach for managing subchondral osteoid osteomas. O-arm navigation with arthroscopic visualization effectively manages subchondral osteoid osteomas by combining surgical excision and CT-guided radiofrequency ablation. This preserves joints, reduces pain, and enables rapid return to function, representing a significant surgical advancement.
